# Pornography use and its association with risk taking sexual behaviours among Irish adolescents

**DOI:** 10.1016/j.puhip.2026.100829

**Published:** 2026-07-17

**Authors:** Nicola M. Murphy, Breeda Neville, Emmet S. Major, Peter M. Barrett

**Affiliations:** a Department of Public Health, HSE-Southwest, Cork, Ireland; b Royal College of Physicians of Ireland, Dublin, Ireland; c National Cancer Control Programme, Dublin, Ireland; d Western Regional Drug and Alcohol Task Force, Galway, Ireland; e School of Public Health, University College Cork, Cork, Ireland; f INFANT Research Centre, University College Cork, Cork, Ireland

**Keywords:** Pornography, Adolescent, Sexual health

## Abstract

**Objectives:**

Online pornography is increasingly accessible to European teenagers due to the proliferation of mobile phone ownership. This raises concerns regarding the influence that pornography may have on adolescent sexual health behaviours. This study examines the association between pornography use and potential risk-taking sexual behaviour among adolescents in the West of Ireland.

**Study design:**

This is a cross-sectional study design, comprising secondary data analysis of the third Planet Youth Survey in the West of Ireland carried out in November 2022 and based on the international Planet Youth Survey.

**Methods:**

A cross sectional analysis was conducted using anonymised data from the third wave of the Planet Youth Survey (November 2022), involving 5277 adolescents aged 13-17 from all post-primary schools and Youthreach centres in Galway, Mayo and Roscommon in Ireland. Exposure variables included recent pornography use, use of pornography as a sexual health information source, and reported pressure to view pornography. Outcome variables included engaging in condomless sex, sexual activity under the influence of alcohol or drugs (in the previous year) and very early initiation of sexual activity (<15 years). Data were analysed using multivariable-adjusted logistic regression.

**Results:**

Thirty-seven percent of respondents reported using pornography in the previous year, with a higher prevalence (51%) among males. Pornography use was significantly associated with engaging in condomless sex (aOR 3.38, 95% C.I. 2.60-4.43), sexual activity under the influence of alcohol/drugs (aOR 3.60, 95% C.I. 2.97-4.37) and very early sexual initiation (aOR 2.79, 95% C.I. 2.00-3.91). Higher levels of parental monitoring consistently conferred a protective effect across all three outcomes.

**Conclusions:**

Pornography use is prevalent among adolescents in Ireland and is associated with risk-taking sexual behaviours. While causality cannot be inferred, these findings support the prioritisation of integrated, evidence-based sexual health strategies incorporating parental engagement, comprehensive school-based sexuality education and broader public health interventions to address risk-taking sexual behaviours among adolescents.

## 1 Introduction

Formative experiences during adolescence can impact on long-term sexual health and well-being, yet inconsistent and unreliable sexual health education often leads adolescents to seek information from online pornography [[1], [2], [3]]. This can result in adolescents modelling behaviours observed in explicit content and potentially normalising risk-taking sexual behaviours [4]. No internationally accepted definition of pornography exists, and the prevalence of its use among adolescents varies widely in the literature due to inconsistent definitions [5]. Despite this, it is likely that adolescent pornography use has increased compared to previous generations due to technological advances, including easier access to explicit content online among teenagers [5,6].

Pornography use is associated with the duration of time spent online [7]. In Ireland, a cross-sectional survey reported that children first access the internet at 6-7 years, with the majority spending 1-2 h per day online, and with increasing time spent online as age advances [8]. In 2023, a UK study reported that 64% of young people aged 16-21 years viewed online pornography, with 10% of this cohort having viewed it by age 9, and first exposure occurring on average at age 13 years [4]. While pornography use appears to be widespread, it is reported to be more prevalent among males, and among those with lower levels of parental monitoring or intergenerational communication [5,7,9].

Pornography use has been linked with engagement in risky sexual practices among adolescents, including earlier sexual initiation and condomless sex [3,10]. According to a national cross-sectional study on pornography use among 20 year olds in Ireland, both male and female pornography users reported engaging in less consistent condom use than those who did not use pornography. However, this survey did not provide insights on the impact of pornography among the younger adolescent population [9].

Sexual assertiveness—the ability to communicate boundaries, express sexual preferences, and insist on condom use—plays a key preventative role in non-consensual sex [11,12]. Sexual health education can play an important role in improving sexual assertiveness, increasing consistent condom use, and reducing adolescent pregnancy and sexually transmitted infection (STI) acquisition [11,13,14]. However, the effectiveness of sexual health education may vary, depending on its content and source [15]. US studies show that parental communication and open discussion about sexual health are linked to delayed sexual initiation and higher contraceptive use among teenagers [15,16], whereas reliance on peers for sexual health information is associated with riskier sexual behaviours, including earlier sexual initiation and lower contraceptive use [15].

While some sources argue in favour of potential educational benefits of pornography use [17,18], systematic and meta-analytic evidence highlights a range of adverse outcomes, including reinforcement of harmful norms, risky sexual practices and associations with sexual aggression [19,20]. Early sexualisation of adolescents can have a significant impact on long term wellbeing and sexual behaviour [4]. Given the likelihood of increasing access to online pornography among adolescents, there is a need for further research on its impact on this vulnerable population, including on their sexual risk-taking behaviours. This study aims to investigate the association between pornography use and risk-taking behaviours, including very early sexual initiation, among adolescents in the West of Ireland.

## 2 Methods

### 2.1 Study design

Data was examined from the third wave of the Planet Youth Survey, which is an anonymous questionnaire completed by over five thousand students from post-primary education settings in counties Galway, Mayo and Roscommon. This 92 item questionnaire includes questions on health and wellbeing, family and school experience, leisure activities, internet and screen use, substance use and sexual health, among others [21].

### 2.2 Study population

The target population comprised students aged 13-17 years from all post-primary schools and Youthreach (the national education and training programme for early school leavers) centres across three counties in the West of Ireland (n = 6095). All 81 post-primary schools and 10 Youthreach centres agreed to participate. Information leaflets regarding the survey were provided to students and parents, with passive consent allowing an opt out. A total of 5277 survey responses were collected, yielding an overall response rate of 86.6%. A small number of students opted out of the process (n = 40) and the remainder (n = 778) were unavailable on the day, due to absenteeism.

### 2.3 Data collection

Data were collected in November 2022 using the Planet Youth Survey Questionnaire using computer-assisted self-interviews (CASI) on school devices via the Alchemer platform. Data was processed in the Icelandic Centre for Social Research Analysis (ICRSA) in Reykjavik for quality check and cleaning of data, and returned to Ireland in February 2023.

### 2.4 Exposure variables

Pornography use was included as the main exposure of interest. Three questions relating to previous pornography use were included: how often, in the past year, participants had (i) viewed pornography (primary exposure) (ii) used pornography as a source of information to learn about sex, and (iii) felt pressured to view pornography by a sexual partner or peer group. Responses were coded as binary variables and classified as never or ever (i.e. at least once in the last 12 months).

### 2.5 Outcome variables

Key outcome variables identified were: (i) engaging in condomless sex in the past year (ii) engaging in sexual activity under the influence of alcohol or drugs in the past year, and (iii) very early sexual initiation among adolescents. No universally accepted definition exists for ‘early sexual initiation’. We defined very early sexual initiation as ever having engaged in sexual intercourse before age 15 years in accordance with the United Nations Programme on HIV/AIDS (UNAIDS) [22].

### 2.6 Confounders

Potential confounding variables were identified from the survey and those selected for inclusion were decided based on a scoping literature review, and grouped into relevant domains for analysis**.** Socio-demographic variables included age, gender, ethnicity, maternal education and deprivation. There was no direct question in the survey regarding family income, therefore a proxy measure for deprivation was derived from participants’ agreement with the statement “my parents/carers hardly have enough money to pay for necessities”.

Variables related to parental influence included measures of parental monitoring and rule-setting. A variable for ‘parental monitoring’ was created by combining participants' responses to the question: “How well do the following statements apply to you?” (i) “My parents/carers know where I am in the evening” and (ii) “My parents/carers know who I am with in the evening”. These statements were selected based on findings from previous studies indicating that higher levels of parental monitoring may be associated with a lower likelihood of pornography use among adolescents [9,23]. Responses were categorised as ‘higher level of parental monitoring’ if the respondents agreed with one or more of the statements, that their parents either knew where they were in the evening, or who they were with in the evening. We considered participants to have a ‘lower level of monitoring’ if they disagreed with both statements and therefore had stated that their parents neither knew where they were, nor who they were with, in the evening. Similarly, a combined variable was created for ‘parental rule-setting’ by combining responses to the question: “How well do the following statements apply to you?” (i) “My parents/carers set rules about what I can do at home” and (ii) “My parents/carers set rules about what I can do outside the home”. Respondents were considered to have a ‘higher level of parental rule-setting’ if they agreed with one or more of the statements that their parents either set rules around what they could do inside the home, or outside the home, but they were considered to have ‘lower level of parental rule-setting’ if they disagreed with both statements, and their parents did not set rules for them either inside or outside the home.

A variable was created relating to self-reported body image among adolescents based on associations from the literature around negative body image and pornography use [9]. This was created by combining responses to the statements “I think that I am ugly and unattractive” and “I am unhappy with my body”. Responses were re-categorised into ‘poor self-image’ if participants agreed with one or more of these statements. While the survey included several items relating to self-image, these items were selected as they most directly captured respondents' current perceptions of their physical appearance. Participants were considered to have ‘good self-image’ if they disagreed with both statements.

Finally, a new variable was created regarding receipt of sexual health information from a ‘trusted source’ based on their responses to statements around (i) having received sexual health information in school, (ii) having spoken to their parents regarding their sexual health or (iii) having spoken to a medical professional regarding their sexual health in the past year. These three items were the only survey questions assessing the receipt of sexual health information. As they captured information received from three trusted sources, they were combined to create a composite variable representing the receipt of sexual health information from trusted sources. Responses were categorised as never having received sexual health information from a ‘trusted source’ (in the last 12 months), if they had not received advice regarding their sexual health from any of the three sources, and therefore disagreed with all three statements. They were considered to have received sexual health advice from a ‘trusted source’ if they agreed that they had received advice from at least one of these sources in the previous year.

### 2.7 Statistical analysis

Data analysis was conducted using STATA version 15.1 (StataCorp LP, College Station, Texas, USA) with significance level set at p < 0.05. Descriptive analysis was performed on the exposure and outcome variables and bivariate associations were investigated using Chi-Squared (χ^2^) tests. Multivariable logistic regression was used to explore links between exposure variables and our three respective outcome variables of condomless sex, sex under the influence of alcohol/drugs, and very early sexual initiation, based on complete case analysis. Crude odds ratios (OR) and adjusted odds ratios (aOR) with 95% confidence intervals were reported for all measures of association.

## 3 Results

Table 1 presents the characteristics of the study participants. Over one third (37%) of respondents had viewed pornography in the previous year. The prevalence of viewing pornography increased incrementally with age, with 42% of those aged ≥17 years (n = 270) having viewed it compared with 35% of 15 year olds (n = 515). In total, 51% of males (n = 1315) and almost one quarter of females (n = 570) had viewed pornography in the previous year. Almost 15% (n = 764) of respondents reported using pornography as a source of information to learn about sex, of which 60% (n = 459) were males. Relatively few participants (3%, n = 147) reported feeling pressured to view pornography by a peer/sexual partner in the previous year (Table 1). Almost 44% of respondents reported the age at which they first watch pornography. Almost one fifth (18%) of respondents watched pornography for the first time aged ≤12 years, while a further 11% reported having watched it for the first time aged ≤13 years, and a further 8% at ≤14 years.

**Table 1:** Characteristics of the study participants (n = 5277).

Variables	N	%
**Age (years)**		
*≤14*	14	0.3
*15*	1462	27.7
*16*	3153	59.7
*≥17*	646	12.2
**Gender**		
*Male*	2577	48.8
*Female*	2540	48.1
*Other/Unknown*	151	2.9
**Ethnicity**		
*White*	4628	87.7
*Non-white*	641	12.1
**Maternal education**		
*Third level*	3426	64.9
*No third level*	1394	26.4
*Unknown*	457	8.7
**Deprivation**^a^		
*Rarely*	4461	84.5
*Sometimes*	231	4.4
*Often*	478	9.1
**Parental rules**^b^		
*Higher level*	4166	78.9
*Lower level*	1009	19.1
**Parental monitoring**^c^		
*Higher level*	4927	93.4
*Lower level*	262	5.0
**Self-image**^d^		
*Poor image*	2873	54.4
*Good image*	2176	41.2
*Unknown*	258	4.9
**Trusted source**^e^		
*Never*	2773	52.5
*Ever*	1940	36.8
*Unknown*	564	10.7
**Viewed porn (last 12 months)**		
*Never*	2755	52.2
*Ever*	1971	37.4
*Unknown*	551	10.4
**Felt pressured to view porn (last 12 months)**		
*Never*	4584	86.9
*Ever*	147	2.8
*Unknown*	546	10.3
**Used porn as a source of information (last 12 months)**		
*Never*	3977	73.4
*Ever*	764	14.5
*Unknown*	536	10.2
**Condomless sex (last 12 months)**		
*Never*	4410	83.6
*Ever*	341	6.5
*Unknown*	526	10.0
**Sex under the influence (last 12 months)**		
*Never*	3997	75.7
*Ever*	735	13.9
*Unknown*	545	10.3
**Very early sexual initiation (<15 years)**		
*Never*	4532	85.9
*Ever*	217	4.1
*Unknown*	528	10.0

a The statement “my parents hardly have enough money to pay for necessities” was used as a marker for deprivation.

b Parental rules refer to “my parents set rules for what I can do in-/outside the home”.

c Parental monitoring refers to “my parents know where I am/who I am with in the evenings”.

d Self-image refers to “I think I am ugly/I am unhappy with my body”.

e Sexual health information refers to whether the respondent spoke a parent, medical professional or teacher regarding their sexual health in the previous 12 months.

### 3.1 Condomless sex

Condomless sex in the previous year was reported by 6.5% of respondents overall, or 46% of sexually initiated adolescents (n = 341). Adolescents whose mother had a third level education were less likely to have engaged in condomless sex (aOR 0.63 95% C.I. 0.49-0.82), whereas adolescents who experienced deprivation were more likely to have engaged in condomless sex. Adolescents reporting higher levels of parental monitoring were less likely to have engaged in condomless sex (aOR 0.48 95% C.I. 0.32-0.71) (Table 2).

**Table 2:** Adjusted analysis for pornography use and engagement in condomless sex, sex under the influence of alcohol/drugs and very early sexual initiation in the last 12 months.

*Variables*	Condomless sex (n = 4751)		Sex under influence (n = 4732)		Very early sex initiation (n = 4749)	
	*Adjusted OR (95% CI)*	*p*	*Adjusted OR (95% CI)*	*p*	*Adjusted OR (95% CI)*	*p*
**Viewed Pornography (last 12 months)**						
Never	Ref		Ref		Ref	
Ever	3.38 (2.60-4.43)	<0.001	3.60 (2.97-4.37)	<0.001	2.79 (2.00-3.91)	<0.001
**Age**						
≥17	Ref		Ref		Ref	
16	0.40 (0.30-0.54)	<0.001	0.53 (0.42-0.67)	<0.001	0.57 (0.38-0.85)	<0.001
15	0.34 (0.24-0.45)	<0.001	0.42 (0.32-0.55)	<0.001	0.81 (0.52-1.25)	<0.001
≤14	1.29 (0.26-6.38)	0.76	0.74 (0.15-3.76)	0.72	-	
**Gender**						
Other	Ref		Ref		Ref	
Male	1.14 (0.59-2.22)	0.69	1.19 (0.71-1.98)	0.51	0.80 (0.38-1.66)	0.55
Female	1.64 (0.84-3.18)	0.15	2.37 (1.42-3.95)	0.001	0.97 (0.46-2.02)	0.93
**Ethnicity**						
White	Ref		Ref		Ref	
Non-white	1.05 (0.73-1.50)	0.79	0.54 (0.39-0.74)	<0.001	1.36 (0.91-2.04)	0.14
**Maternal Education**						
No third level	Ref		Ref		Ref	
Third level	0.63 (0.49-0.82)	0.001	0.87 (0.72-1.06)	0.16	0.63 (0.45-0.87)	0.006
**Deprivation**						
Rarely	Ref		Ref		Ref	
Sometimes	1.61 (1.01-2.56)	0.04	1.09 (0.74-1.60)	0.68	2.23 (1.33-3.74)	0.002
Often	1.52 (1.04-2.22)	0.03	0.89 (0.64-1.22)	0.47	1.41 (0.88-2.26)	0.16
**Parental rule setting**						
Lower level	Ref		Ref		Ref	
Higher level	0.84 (0.63-1.10)	0.20	1.02 (0.83-1.26)	0.84	0.99 (0.69-1.40)	0.94
**Parental monitoring**						
Lower level	Ref		Ref		Ref	
Higher level	0.48 (0.32-0.71)	<0.001	0.29 (0.21-0.40)	<0.001	0.39 (0.24-0.61)	<0.001

*Variables*	Condomless sex (95% CI) (n = 4751)		Sex under influence (95% CI) (n = 4732)		Very early sex initiation (95% CI) (n = 4749)	
	*Adjusted OR (95% CI)*	*p*	*Adjusted OR (95% CI)*	*p*	*Adjusted OR (95% CI)*	*p*
**Self-image**						
Poor	Ref		Ref		Ref	
Good	0.85 (0.66-1.09)	0.19	0.75 (0.63-0.90)	0.002	0.93 (0.68-1.28)	0.67
**Trusted source of sexual health information**						
Never	Ref		Ref		Ref	
Ever	1.33 (1.04-1.69)	0.02	1.47 (1.23-1.74)	<0.001	1.26 (0.94-1.73)	0.12
**Parental Source**						
Never	Ref		Ref		Ref	
Ever	2.01 (1.52-2.65)	<0.001	1.42 (1.13-1.74)	0.002	1.80 (1.27-2.55)	0.001
**School Source**						
Never	Ref		Ref		Ref	
Ever	1.08 (0.85-1.38)	0.53	1.27 (1.07-1.51)	0.007	0.97 (0.71-1.32)	0.85
**Medical Source**						
Never	Ref		Ref		Ref	
Ever	4.05 (2.90-5.66)	<0.001	2.10 (1.56-2.84)	<0.001	3.10 (2.04-4.72)	<0.001

*The statement “my parents hardly have enough money to pay for necessities” was used as a marker for deprivation. †Parental rules refer to “my parents set rules for what I can do in-/outside the home”. ‡Parental monitoring refers to “my parents know where I am/who I am with in the evenings”. § Self-image refers to “I think I am ugly/I am unhappy with my body”. ¶ Sexual health information refers to whether the respondent spoke a parent, medical professional or teacher regarding their sexual health in the previous 12 months.

Those who viewed porn, felt pressured to view porn, or used porn as a source of information to learn about sex in the previous 12 months were two to three times more likely to have engaged in condomless sex compared to those who had not (aOR 3.38 95% C.I. 2.60-4.43, aOR 3.66 95% C.I. 2.37-5.63, and aOR 2.54 95% C.I. 1.96-3.32 respectively) (Table 2, Table 3). Of note, those who had spoken to a parent or a medical source regarding their sexual health, were more likely to have engaged in condomless sex in the previous year (aOR 2.01 95% C.I. 1.52-2.65, and aOR 4.05 95% C.I. 2.90-5.66 respectively).

**Table 3:** Unadjusted and adjusted odds ratios of outcomes of condomless sex, sex under the influence and very early sexual initiation with exposure variables of using porn as a source of information and feeling pressured to view porn.

*Exposure Variables*	Condomless sex (n = 4751)		Sex under influence (n = 4732)		Very early sexual initiation (n = 4749)	
	*Unadjusted OR* (95% CI)	*Adjusted OR* (95% CI)	*Unadjusted OR* (95% CI)	*Adjusted OR* (95% CI)	*Unadjusted OR* (95% CI)	*Adjusted OR* (95% CI)
**Porn as a source of information**	3.00 (2.35-3.81)	2.54 (1.96-3.32)	2.77 (2.31-3.32)	2.48 (2.03-3.03)	2.27 (1.67-3.09)	1.90 (1.35-2.67)
**Pressured to view porn**	4.13 (2.76-6.19)	3.66 (2.37-5.63)	3.41 (2.41-4.84)	3.00 (2.07-4.35)	3.86 (2.38-6.27)	3.1 (1.77-5.13)

Unadjusted and adjusted odds ratios of exposures viewed pornography, used porn as a source of information to learn about sex and sex under the influence (last 12 months) with outcomes condomless sex, sex under the influence and very early sexual initiation adjusted for confounders age, sex, ethnicity, maternal education, deprivation, parental factors, self-image, sexual health information.

### 3.2 Sexual activity under the influence of alcohol or drugs

Over 27% of respondents reported engaging in sexual activity in the previous year. Almost 14% (n = 735) reported engaging in sexual activity under the influence of alcohol/drugs in this timeframe (Table 1). Adolescents who reported greater levels of parental monitoring were significantly less likely to have to have engaged in sexual activity under the influence (aOR 0.29, 95% C.I. 0.21-0.40) (Table 2).

Those who had viewed pornography, used porn as a source of information to learn about sex, and those who felt pressured to view porn were two to three times more likely to engage in sexual activity under the influence of alcohol or drugs (aOR 3.60, 95% C.I. 2.97-4.37, aOR 2.48 95% C.I. 2.03-3.03 and aOR 3.00, 95% C.I. 2.07-4.35), compared with those who had not (Table 2, Table 3). Adolescents reporting better self-image were less likely to have engaged in sexual activity under the influence (aOR 0.75, 95% C.I. 0.63-0.90). Notably, those who had spoken to a trusted source regarding their sexual health were more likely to have had sexual activity under the influence of alcohol/drugs in the previous 12 months (aOR 1.47 95% C.I. 1.23-1.74), and this finding persisted when these sources (i.e. parent, school teacher, or medical professional) were examined separately (Table 2). However, it was not possible to examine the temporal nature of any of these associations.

### 3.3 Very early sexual initiation

Among the respondents who were sexually initiated (n = 740), 29% of them (n = 217) were aged <15 years at sexual initiation. Adolescents whose mothers had received a third level education were less likely to have had very early sexual initiation (aOR 0.63 95% C.I. 0.45-0.87). Those who sometimes experienced deprivation were more likely to have had very early sexual initiation (aOR 2.23 95% C.I. 1.33-3.74). Adolescents reporting higher levels of parental monitoring were less likely to report very early sexual initiation (aOR 0.39, 95% C.I 0.24-0.61) (Table 2).

Having viewed pornography, used pornography as a source of information to learn about sex, or having felt pressure to view pornography by a partner/peer were all significantly associated with very early sexual initiation (aOR 2.79 95% C.I. 2.00-3.91, aOR 1.90 95% C.I. 1.35-2.67, and aOR 3.01 95% C.I. 1.77-5.13 respectively) (Table 2, Table 3).

## 4 Discussion

Over 37% of study participants had viewed pornography in the last year, including over half of male respondents and almost one quarter of females. Pornography use was more prevalent among males and increased incrementally with age. Almost 15% of teenagers reported using pornography as a source of information to learn about sex, while relatively few (<3%) felt pressured to view pornography by a peer or sexual partner. Over one third of respondents (37%) reported first watching pornography before 14 years. Pornography use was consistently associated with a higher likelihood of engaging in risk-taking sexual behaviours. Although parental rule setting did not impact significantly, higher levels of parental monitoring were associated with reduced risk-taking sexual behaviours among adolescents.

Although prevalence estimates vary, there is broad consensus that adolescent pornography use has increased compared to previous generations [5]. The prevalence reported in our study (37% last year) is lower than that reported in earlier research [6,24,25], which may reflect the younger age profile of our study sample (predominantly 15-16 years), whereas prior studies included older adolescents who typically report higher rates of use. Variations in geographical and cultural context may also contribute [7,24,25]. Previous observational studies have indicated that pornography use is more prevalent among males from more affluent backgrounds [5,6,9]. Although our study employed a proxy for socioeconomic status of participants, pornography use was nevertheless found to be more prevalent among males.

The initiation of sexual activity can involve risk taking behaviours such as inconsistent use of condoms/contraceptives, and subsequent increased risk of STIs or unplanned pregnancy, among other adverse outcomes [26]. We found that 46% of those who were sexually initiated reported condomless sex at least once in the previous year. An Irish study of adolescents from 2018 reported a lower prevalence of condomless sex (20%), although this was based solely on condom use at most recent intercourse [27]. In our study, adolescents who viewed pornography were three times more likely to engage in condomless sex, which is consistent with an Irish cross sectional study of young adults and a nationally representative survey of young adults from the USA [9,10]. Although causal associations cannot be deduced, this raises questions about the potential influence that the pornography industry may have on teenagers through its own depiction of condomless sex.

Evidence from U.S. adolescent populations indicates that parental provision of sexual health information around sexual health is associated with safer sexual behaviours, including higher contraceptive use [15,28]. While our study did not assess female contraceptive use, higher levels of parental monitoring were associated with lower odds of engagement in condomless sex. Unexpectedly, adolescents receiving sexual health information from parents or medical sources had higher odds of condomless sex, contrasting with U.S. findings that parent–adolescent communication reduces such behaviour among pornography users [29]. This discrepancy may reflect recall or social desirability bias, previously described when assessing sensitive behaviours among adolescents [30]. Alternatively it may be explained by the cross-sectional design, which precludes determination of temporal relationships; participants may have engaged in condomless sex before receiving advice. These findings also raise questions regarding the effectiveness of sexual health education provided by trusted sources and underscore the importance of developing porn literacy as a means of facilitating meaningful communication with adolescents. Recent revisions to Ireland's Relationship and Sexuality Education curriculum and the National Sexual Health Strategy 2025-2035 emphasise the need for a structured educational approach to addressing the impact of pornography on young peoples' expectations of sex and relationships [31]. In the Irish context, an integrated approach should align with the National Sexual Health Strategy by embedding age-appropriate porn literacy within comprehensive sexual health education, supported by resources for parents and teachers and reinforced through coordinated school, youth and public health initiatives.

In Ireland, adolescent heavy episodic drinking (HED) rates are similar to that of our European counterparts [32], while cannabis and cocaine use are higher than the European average [33,34]. Our study found pornography use was associated with increased likelihood of sexual activity under the influence of alcohol or drugs. While similar associations have been reported in some adolescent studies, the evidence remains limited and is based predominantly on cross-sectional studies [35,36]. This association may reflect a broader clustering of risk-taking behaviours, potentially influenced by shared individual, social and environmental factors. This finding is concerning, given the association between adolescent substance use and risky sexual behaviours [37]. Teenagers who had received sexual health advice in the previous year were more likely to have engaged in sexual activity under the influence, a finding that contrasts with US longitudinal evidence reporting that enhanced sexual health education was associated with a lower likelihood of using drugs or alcohol prior to sex [38]. Again, as the study is cross-sectional, it is possible that substance-related sexual activity preceded receipt of the sexual health advice.

Early sexual initiation can be linked with adverse outcomes including adolescent pregnancy and STI acquisition [26]. Adolescents who viewed pornography in the previous year were more than twice as likely to have had very early sexual initiation compared with those who had not. This was consistent with the findings of a previous systematic review, and with an Irish study of older adolescents [1,9]. We found that very early sexual initiation appeared to be socially patterned; this aligns with findings of a large US cross-sectional study of male high-school students, in which maternal education was protective against early sexual initiation [23].

Adolescents exposed to higher levels of parental monitoring were less likely to report very early sexual initiation, and the protective influence of parental monitoring was consistently observed across all sexual risk-taking behaviours in this study. This contrasts with a cross-sectional study of Croatian adolescents which did not report any protective effect between parental monitoring and teenagers’ risk taking sexual behaviours [39]. Although this study was comparable to ours in terms of respondent age range (14-17 years), and its cross-sectional design, our findings were based on substantially a larger sample size which strengthens the validity of our results.

Strengths of this study include the use of a large, robust dataset which was quality-checked and cleaned following international standards [21], a large sample size, with high levels of participation and high levels of data completeness. The survey was self-administered and anonymous, which minimised inherent bias. However, several limitations should be noted. The self-reported nature of the survey may introduce social desirability bias, particularly regarding sensitive behaviours among adolescents [30]. Some variables were combined based on participants’ broad agreement or disagreement with multiple statements; while this clarified overall patterns from a public health perspective, it may have reduced statistical variability, and a more nuanced analysis may have added some additional context. The use of a proxy measure for deprivation was necessary, as the survey did not include a direct measure of household income. However, reliance on this proxy may introduce measurement bias, given the complex and multifactorial nature of socioeconomic status. As in similar studies, the absence of a standardised definition of pornography use may have led to varied interpretations, limiting direct comparison with other research.

This study provides novel insights on the prevalence of pornography use among a large sample of adolescents in Ireland. Pornography use was found to be associated with a two to three fold likelihood of engaging in risk taking sexual behaviours, including inconsistent condom use, sex under the influence of alcohol or drugs, and very early sexual initiation. Our findings suggest the need for a collaborative approach to sexual health education including an emphasis on porn literacy for parents, teachers, and adolescents alike.

## Ethical statement

Ethical approval was granted for the Planet Youth Survey 2022 by the Royal College of Physicians of Ireland. All data examined was anonymised at point of receipt and presented as such. Access to study data was password protected and only accessible to the author. GDPR legislation was adhered to by the author throughout.

## Funding

No external funding was obtained to complete this work.

**Key points**

• Over one third of Irish adolescents reported using pornography in the past year, with usage higher amongst males (51%) than females (24%).
• Pornography use was strongly associated with engaging in risk-taking sexual behaviours of condomless sex, sexual activity under the influence of alcohol or drugs and very early sexual initiation (<15 years).
• Higher levels of parental monitoring consistently reduced the likelihood of engaging in risk-taking sexual behaviours.
• Findings highlight the need for integrated, evidence-based sexual health strategies, that include parental involvement, school based education and broader public health interventions.

## Declaration of competing interest

I certify that I have no affiliations with or involvement in any organisation or entity with any financial interest (such as honoraria; educational grants; participation in speakers’ bureaus; membership, employment, consultancies, stock ownership, or other equity interest; and expert testimony or patent-licensing arrangements), or non-financial interest (such as personal or professional relationships, affiliations, knowledge or beliefs) in the subject matter or materials discussed in this presentation/study.
